# Gαo (*GNAO1*) encephalopathies: plasma membrane *vs*. Golgi functions

**DOI:** 10.18632/oncotarget.22067

**Published:** 2017-10-25

**Authors:** Gonzalo P. Solis, Vladimir L. Katanaev

**Affiliations:** Vladimir L. Katanaev: Department of Pharmacology and Toxicology, University of Lausanne, CH-1011 Lausanne, Switzerland; School of Biomedicine, Far Eastern Federal University, Vladivostok, Russian Federation

**Keywords:** heterotrimeric Gαo, *GNAO1* encephalopathy, GPCR, Golgi apparatus

Heterotrimeric G proteins are key signaling molecules best recognized as the immediate transducers of GPCRs (G protein coupled receptors). A heterotrimeric G protein complex consists of three subunits, α, β, and γ, of which the Gα is responsible for binding to guanine nucleotides and to the cognate GPCR. Ligand-activated GPCRs catalyze the GDP-GTP exchange on Gα inducing the dissociation of Gα-GTP from Gβγ, both being competent to engage downstream effectors. GTP hydrolysis returns the Gα to its GDP-bound state allowing the formation of the heterotrimeric G protein, which binds the cognate GPCR for a subsequent activation cycle.

During the last five years, whole-exome sequencing of patients with severe infantile encephalopathies resulted in an avalanche of *de novo* heterozygous mutations in *GNAO1* [1]. *GNAO1* encodes for Gαo, the major Gα subunit in the mammalian brain. Gαo is one of the sixteen human α-subunits and transduces the signal of a group of rhodopsin-like GPCRs including adrenergic, dopaminergic, opioid, and muscarinic receptor subtypes [2]. Gαo knockout (KO) mice showed a strong developmental delay during the first 3 weeks after birth, multiple neurological abnormalities such as hyperalgesia, hyperactivity, generalized tremor with occasional seizures, and a severe impairment of motor control [2]. At the cellular level, dorsal root ganglion cells derived from Gαo KO mice presented a reduced inhibition of Ca^2+^ channel currents by the activation of opioid receptors. Gαo is additionally implicated in the regulation of Ca^2+^ and K^+^ channels in sensory and hippocampal neurons [2].

It has been proposed that Gαo encephalopathies possess a strong genotype-phenotype association, with a group of mutations causing developmental delay and movement disorders, and another group responsible for the more severe cases of epileptic encephalopathy and Ohtahara syndrome [3]. Thus far, the vast majority of the cases is associated to single point mutations in 16 highly conserved residues [1], pointing to their involvement in basic Gαo functions. The remaining two cases correspond to a deletion of seven amino acids and a predicted splicing defect [1]. A recent study indicates that mutations in eight of the 16 residues cause varying degrees of loss-of-function downstream of the α2A adrenergic receptor [4]. Somehow contradictory, a previous study showed that two of these residues do not induce any significant defects compared to wild type Gαo in the inhibition of Ca^2+^ currents by norepinephrine [5]. Intriguingly, mutations in other five residues did not compromise (or only slightly affected) α2A adrenergic receptor signaling [4]. Thus, it is highly plausible that Gαo functions beyond its canonical role as GPCR-transducer also contribute to the development of Gαo encephalopathies.

**Figure 1:**
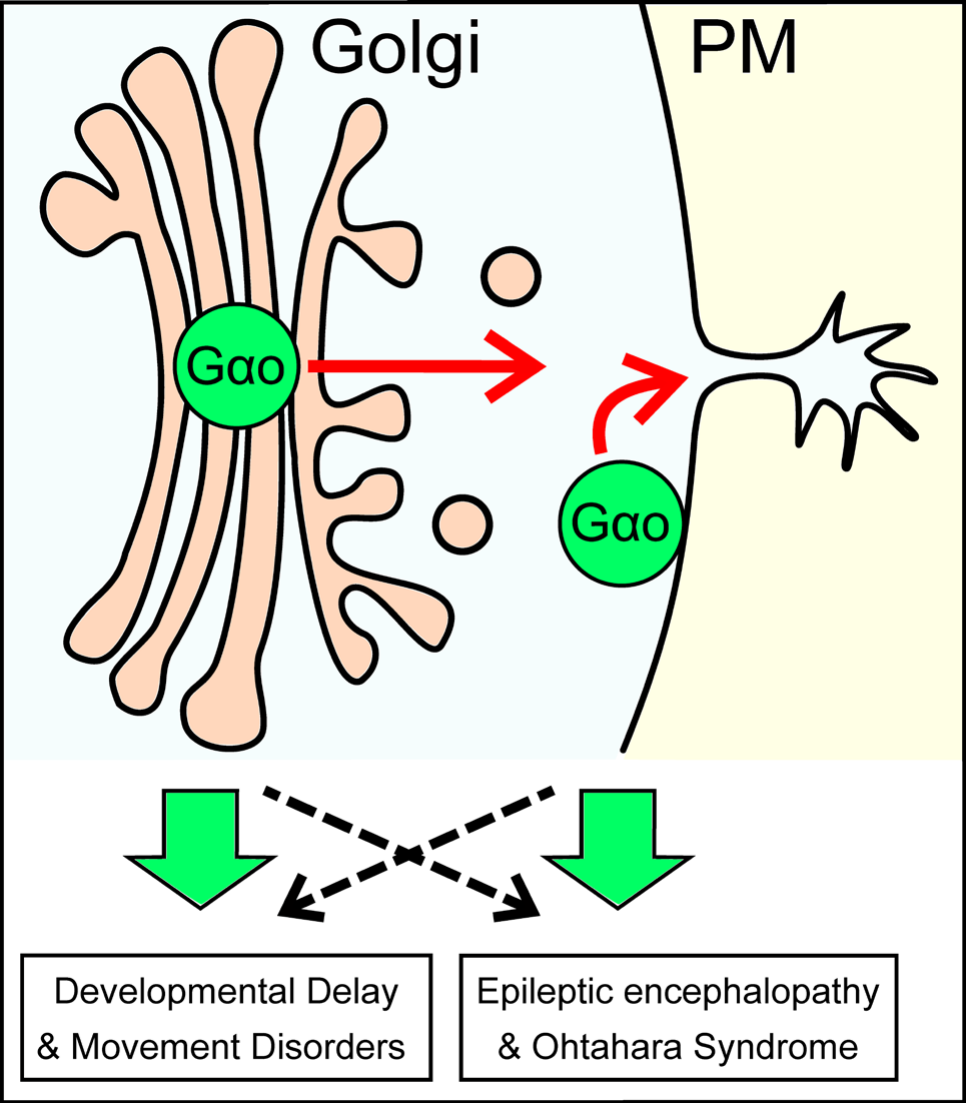
Gαo mutations affecting differently its PM and Golgi functions as the potential origin of the varying degrees of severity observed in *GNAO1* encephalopathies

The functional localization of heterotrimeric G proteins at the plasma membrane (PM) is widely accepted. However, an increasing amount of data indicate that they also work at different cellular compartments, especially at the Golgi apparatus [6]. Recently, we identified >250 proteins as potential Gαo interacting partners, and subsequently uncovered a non-canonical Gαo signaling involved in the regulation of vesicular trafficking at the Golgi [7]. We showed that two pools of Gαo – at the PM and Golgi – coexist and act coordinately in cellular processes such as neuritogenesis: the PM Gαo locally initiates neurite formation, and the Golgi Gαo ensures material delivery for neurites to grow (Figure 1). We nailed down a novel mechanism of unconventional interactions at the Golgi, where an atypical GPCR (KDEL receptor) stimulates Gβγ-free Gαo, which in turn activates the small GTPases Rab1 and Rab3. This pathway is highly conserved from fly to human, and is also required for synaptogenesis *in vivo* [7].

Remarkably, mutations in the Gαo-coupled α2B adrenergic receptor are linked to a familial epilepsy (*ADRA2B*, OMIM:607876 [8]), and several *de novo* mutations in the heterotrimeric Gβ1 are associated to severe neurodevelopmental disorders often accompanied by seizures (*GNB1*, OMIM:616973). On the other hand, some of the Golgi partners of Gαo [7] have been involved in diverse neuropathies characterized by developmental delay and intellectual disability but not seizures (i.e. *COG6*, OMIM:615328; *AP1S1*, OMIM:609313; *GDI1*, OMIM:300849). Therefore, it is tempting to speculate that different degrees of severity in *GNAO1* encephalopathies reflect the extent in which the PM *vs.* Golgi functions of Gαo are affected by a particular mutation (Figure 1). We hypothesize that mutations targeting mainly the canonical GPCR-mediated signaling of Gαo induce the most severe cases of epileptic encephalopathy, whereas mutants affecting predominantly its Golgi role develop neurodevelopmental delay, intellectual disability and movement disorders.

To conclude, the next challenge to eventually complete our understanding of the etiology of *GNAO1* encephalopathies is the generation of a tractable animal model to study the Gαo pathways that control development and physiology of the central nervous system. Such a model should allow for large screening of chemicals to identify hit compounds in order to develop personalized drugs against Gαo encephalopathies.
